# Decomposing delta plots: exploring the time course of the congruency effect using inhibition and facilitation curves

**DOI:** 10.1007/s00426-024-02075-z

**Published:** 2025-02-13

**Authors:** Parker Smith, Rolf Ulrich

**Affiliations:** Fachbereich Psychologie, Eberhard Karls Universität Tübingen, Schleichstr. 4, 72076 Tübingen, Germany

## Abstract

When assessing the time-course of evidence accrual in conflict tasks, delta plots are often employed to show the time course of congruency effects. However, delta plots on reaction time and response errors only capture the differences between the congruent and incongruent conditions, detailing that a pattern or shift is occurring, but not what contributes to creating these changes. To gain a clearer idea of what is causing these trends and shifts, the neutral condition can be added to conflict tasks in order to decompose the congruency effect into two components: facilitation and inhibition. Similarly, the traditional delta plot of the congruency effect can also be decomposed to capture the time-course of facilitation and inhibition in separate curves. Thus, this article endeavored to both assess the utility of inhibition and facilitation curves as a tool for parsing apart the congruency effect, and also to see how the observed patterns changed on a larger time frame. To do this, an exploratory study was conducted on three conflict task experiments (a linguistic flanker task, numeric Stroop task, and symbolic Simon task) that were run with a speed-accuracy tradeoff measure implemented as well. By observing the conflict tasks at various speed stresses, we hoped to evaluate how, or if, inhibition and facilitation change at different response thresholds. The addition of delta functions for facilitation and inhibition provided further insight into base mean RT data. The results also provided evidence for numerous assumptions regarding cognitive control, such as a dominant effect of inhibition driving most of the congruency effect.

## Introduction

Goal-irrelevant information often impairs the cognitive processing of goal-relevant information (e.g., Cohen, 2017; Kornblum et al., 1990; G. A. Miller et al., 1960). Conflict tasks have been utilized to study the mechanisms underlying this impairment. Prototypical examples of these tasks are the Eriksen flanker task (Eriksen & Eriksen, 1974; Eriksen & Schultz, 1979), the Stroop task (Stroop, 1935), and the Simon task (Simon, 1969; Simon & Rudell, 1967). For example, in the Flanker task, participants respond to a target letter (e.g., K) flanked by goal-irrelevant letter information (e.g., HH K HH). When the goal-irrelevant information aligns with the target (e.g., KK K KK), responses are relatively fast and accurate. However, if the goal-irrelevant information signals an alternative response, responses are usually slow and error-prone. The former situation is usually labeled as the *congruent condition*, whereas the latter one as the *incongruent condition*. The difference in response speed between these two conditions is called the *congruency effect*.

In order to analyze the cognitive processes behind the congruency effect, researchers usually utilize stochastic models. A cornerstone of the field is Ratcliff’s decision diffusion model (DDM; (Ratcliff, 1978; Ratcliff & Rouder, 1998; Smith, 2000)). According to this model, a noisy process of information accrual takes place as time evolves. Said information accrual “drifts” towards one of two boundaries, each representing some form of response. Upon reaching a decision boundary, a choice is made, terminating the accrual of information. By fitting the model to the experimental data, researchers can derive implications about the cognitive process by examining the estimated parameters. To accommodate the processes underlying conflict tasks, extensions of Ratcliff’s model have been developed. Examples of these include the *Shrinking Spotlight Model* (SSP; (White et al., 2011)) and the *Diffusion Model of Conflict* (DMC; (Ulrich et al., 2015)).

**Fig. 1:**
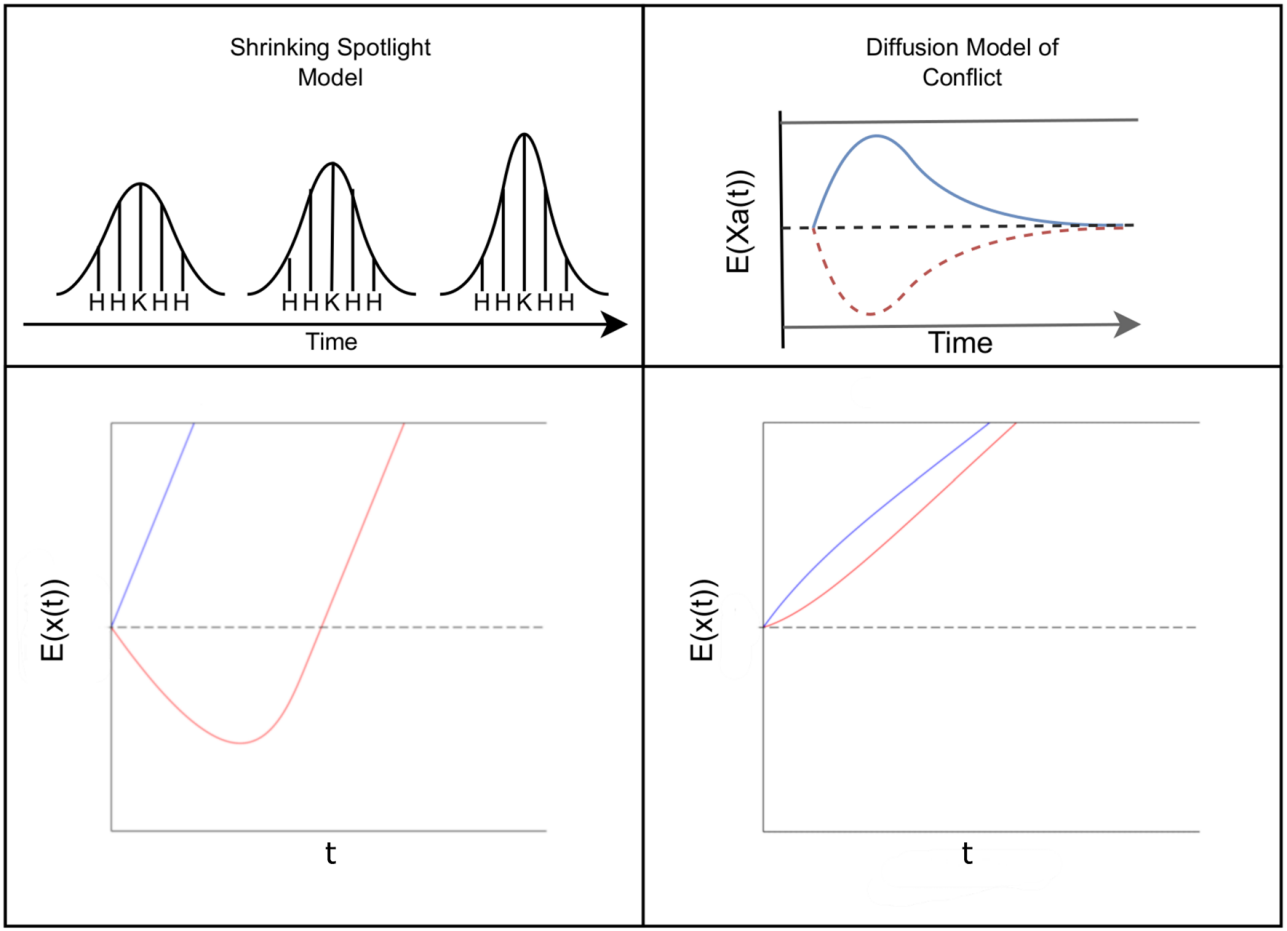
This figure describes a visual interpretation and predictions of the Shrinking Spotlight Model (left) and the Diffusion Model of Conflict (right). For the Shrinking Spotlight Model, the shrinking focus (top left) is displayed such that, over time, the amount of attention given to the flankers is lessened, and instead allotted to the central target. The resulting model is shown below, with blue indicating the congruent condition and red indicating the incongruent condition. For the Diffusion Model of Conflict, the top-right shows the rate of accrual for the automatic processes (red and blue lines) as well as the controlled process (dashed black line). Upon being superimposed, the graph (bottom right) is the result, with congruent and incongruent conditions color-coded the same as with SSP

While Ratcliff’s DDM worked solely off the assumption of a linear accrual of information, SSP’s approach is distinct insofar as that it takes cues from later papers on selective attention. Drawing from the works of early and late selection (Kahneman & Treisman, 1984) and the lens model (Eriksen & St. James, 1986), the model operates by treating visual attention as a process that continuously narrows over time (Fig. 1). Depending on the condition, the drift rate will increase over time as, theoretically, the amount of visual attention attributed towards the target is increased. The continuous nature of this shift is different from its predecessor, the * Dual-Stage Two-Phase model* (Hübner et al., 2010), which utilized a discrete shift in drift rate. In contrast to SSP, DMC attempts to model the two processes of the cognitive control framework, rather than modeling a specific task (Fig. 1). As such, the model focuses on simulating the controlled process (evidence from task relevant information), then overlaying the activation of the automatic process (evidence from the task-irrelevant information) on top of it. The time course of automatic activation is assumed to resemble a pulse function. Accordingly, automatic activation first increases and, after reaching a maximum, dissipates back to zero. This pulse function may reflect that activation of irrelevant information is subject to passive decay (e.g.,Ellinghaus et al., 2018; Hommel, 1994) or to active top-down suppression (e.g.,Ridderinkhof, 2002). For the controlled process, a constant drift rate is utilized, set to favor the correct response. By superimposing the automatic and controlled response functions, one arrives at a time-dependent drift function that initially accelerates faster in favor of the congruent than towards the incongruent conditions (see, Ulrich et al., 2015).

These models are particularly useful for better understanding the time course of conflict processing. Specifically, instead of solely examining the mean reaction time (RT), it is also possible to investigate how the congruency effect evolves over time by analyzing the *delta functions* (often called *delta plots*) (de Jong et al., 1994; Schwarz & Miller, 2012). *Delta functions* provide an established and convenient tool to assess this the evolution of the congruency effect over time. Specifically, these functions evaluate how the congruency effect changes as a function of time (for a comprehensive mathematical description of delta functions, refer to Speckman et al., 2008). Interestingly, the shape of these functions varies across conflict tasks. For example, the flanker and Stroop tasks usually have functions with a positive slope because the congruency effect often grows larger with time (e.g.,Burle et al., 2014; Mittelstädt et al., 2022). In contrast, the Simon effect displays a negative slope because the congruency effect diminishes over time (e.g.,Burle et al., 2014; Mittelstädt & Miller, 2020; Mittelstädt et al., 2022). Within DMC, these different slopes are attributed to different speeds of the automatic process in these conflict tasks. While the underlying reasons for why these trends in congruency effects occur are still mainly the subject of theory, a number of studies have shown that delta plots can offer further information about the effects of experimental manipulations on conflict tasks. Thus, delta plots offer more insight into the data of these experiments versus solely relying on mean RT (e.g., Mittelstädt et al., 2023).

The primary objective of the current study is to scrutinize the temporal progression of the congruency effect in conflict tasks. In particular, the study aims to split apart the congruency effect into its components, inhibition and facilitation, before exploring how they develop. In pursuit of this goal, we integrate two experimental methodologies that have recently been used to examine diffusion models of conflict processing: (a) Incorporating a neutral condition alongside the standard conflict paradigm that typically includes only the congruent and the incongruent condition (Smith & Ulrich, 2023). (b) Adeptly manipulating the trade-off between speed and precision (Mittelstädt et al., 2022). It is hoped that combining these two approaches can enhance our understanding of how congruency effects unfold over time.

**Fig. 2:**
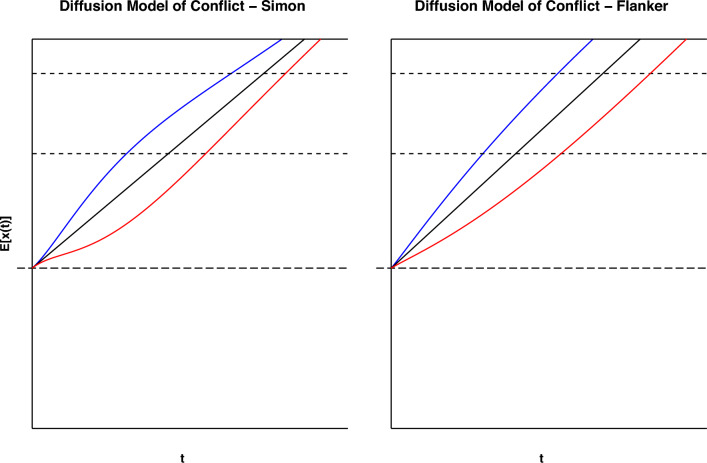
Above is a figure displaying the difference in expected evidence accumulation for DMC between the Simon and flanker tasks. The expected drift rates for the congruent condition (blue) and incongruent condition (red) are shown for each task. Parameter settings for these were taken from Ulrich et al. (2015). While the difference between the rates shrinks in the Simon task, the difference increases for the flanker task, reflecting an expected increase in congruency effect. By adding a neutral condition (black), it is also easy to see how the difference between the three lines changes over time

First, Smith and Ulrich (2023) demonstrated that the inclusion of a neutral condition can provide additional clues about how information accrues in conflict tasks. For example, the neutral condition in a flanker task contains only the target stimulus and uninformative flankers (e.g., SS K SS). The inclusion of a neutral condition enables one to assess whether the mean RT in the congruent or incongruent conditions is closer to the mean RT in the neutral condition. In effect, the addition of this condition ideally allows researchers to decompose the congruency effect into two parts, that is, facilitatory and inhibitiory contributions of the irrelevant information. In particular, facilitation is captured by the difference between the congruent and neutral condition, and represents the gain in performance due to automatic activation, while inhibition is the detriment to performance. Facilitation and inhibition are calculated by taking the differences between the relevant conditions, with the former being the difference between the neutral and congruent conditions, and the latter as the difference between the incongruent and neutral conditions. One can see this in DMC by comparing the difference in expected RT for each concflict task condition in Fig. 2. As is evident, inhibition (the difference between the black and red lines) is greater than facilitation (the difference between the black and blue lines) for faster responses and diminishes over time, irrespective of whether the peak of automatic activation is early (e.g., as in the Simon task) or late (e.g., as in the Flanker task). As argued by Smith and Ulrich (2023), asymmetries in inhibition and facilitation can be diagnostic about how information accrual develops over time.

Second, speed-accuracy manipulation can be also useful when investigating the time-course of conflict processing and the predictions of diffusion models. For example, Mittelstädt et al. (2022) recently showed that mean congruency RT effects increase for the flanker task but decrease for the Simon task under heightened speed stress. As is natural within the framework of diffusion models, they assumed that when participants are instructed to prioritize speed, it would reduce the response boundaries, whereas under accuracy conditions, boundaries increase. Critically, as the Simon effect generally decreases over time (i.e., negative delta plots), and the flanker effect increases with time (i.e., positive delta plots), differences in the peak of automatic activation within DMC can parsimoniously explain the opposing patterns observed in mean RT. Therefore, the manipulation of SAT also provides a potential route to unravel the time-course of congruency effects.

Working from these two studies, the present article computes two separate delta functions for the congruent and incongruent conditions. Specifically, we compare RTs in congruent and neutral trials to generate a delta function that is assumed to capture facilitation, and RTs in the incongruent and neutral conditions to capture inhibition. These two separate facilitation and inhibition curves should trace how the activation accumulates in the two conditions over time. Then, by changing the speed focus of participants, we should be able to raise the response boundary. With this speed-accuracy manipulation, we expect additional insights into how facilitation and inhibition work together to create the congruency effect.

**Fig. 3:**
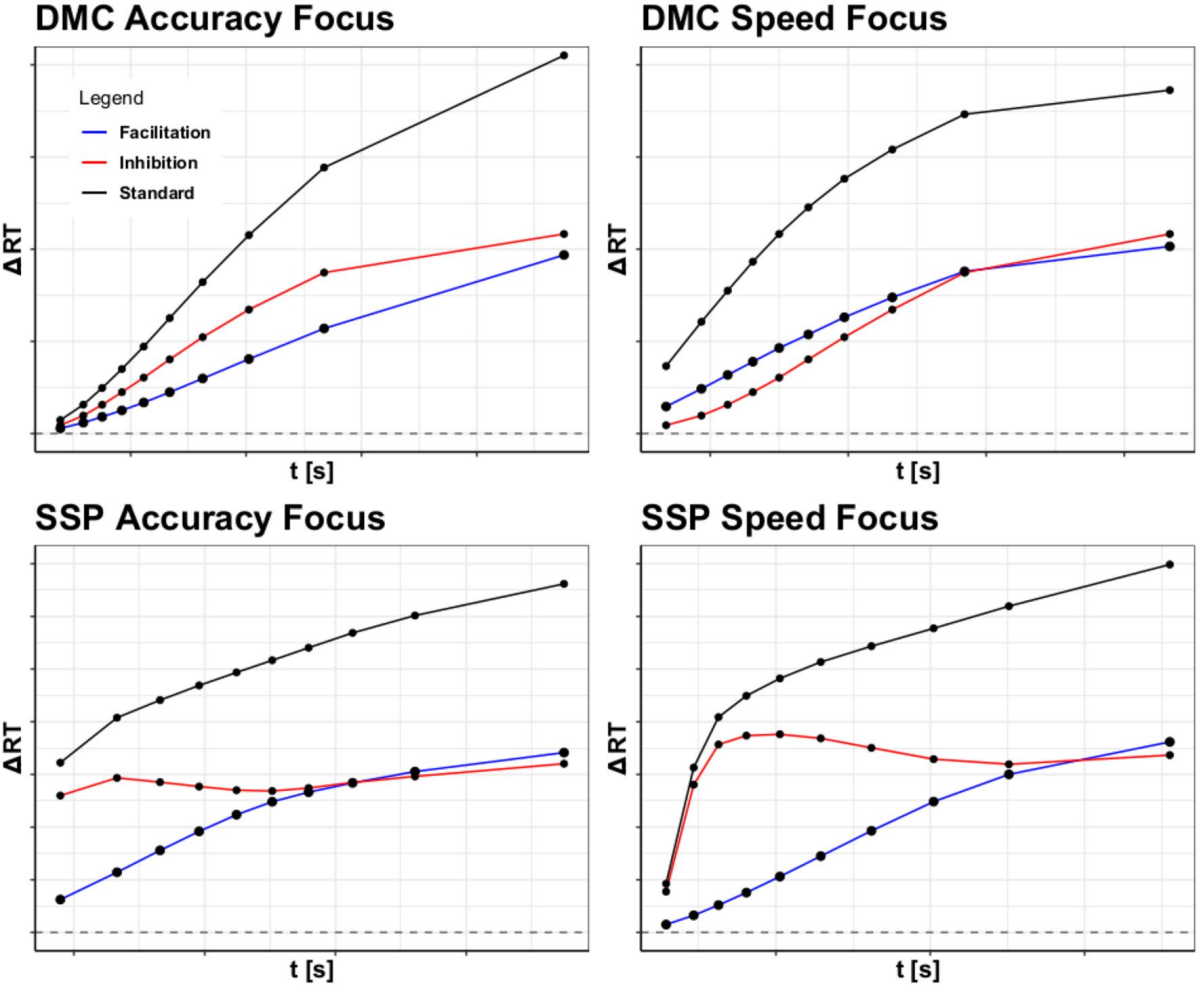
The graphs above display facilitation and inhibition plots for DMC and SSP with different response speed conditions: accuracy-focused responses and speed-focused responses. The simulated inhibition, facilitation, and congruency effects for the accuracy-focused responses are shown on the left, with those for the speed-focused responses shown on the right. Parameter settings were held constant from Fig. 1. These were generated from 100,000 trial simulations for the congruent, neutral, and incongruent conditions. The SAT conditions were manipulated by setting the boundary condition for DMC to 31.30 for speed-focused responses and 71.30 for accuracy-focused responses. Boundary values of 2000 and 4000 were used for SSP. Consult the code resources for further details and scaling

In order to demonstrate the potential diagnostic power of these separate delta functions, we have simulated data with SSP and DMC and computed these two separate delta functions (see Fig. 3). Each delta function was generated using settings for the flanker task found in Ulrich et al. (2015) and White et al. (2011). The resulting plots show that both models predict positive slopes for facilitation, implying an increasing difference between the neutral and congruent conditions. Minor divergences in the models occur in regards to inhibition, with SSP predicting a spike in inhibition before decreasing to a more constant rate, while DMC has a gradual asymptotic prediction. However, both models predict that facilitation and inhibition will eventually become equal. This prediction is made to explain the subsequent increase in congruency effect observed for the flanker and Stroop tasks. Hence, these model-based simulation results demonstrate the potential benefit of gaining additional insights into the mechanisms of conflict processing.

To further test both the model predictions and the utility of this approach, we set up three pre-registered, two-choice conflict task experiments (the linguistic flanker task, the numeric Stroop task, and the symbolic Simon task). These three tasks should, in essence, allow for an examination of both the generality of the models and also how facilitatory- and inhibitory-delta plots vary across different types of conflict tasks In each experiment, we manipulated the trade-off between speed and accuracy by having three conditions (“Speed”, “Accuracy”, and “Balanced”) to raise and lower the response boundary threshold. While the original pre-registered experiments solely relied on mean RT, the delta function analysis was considered to be a potentially fruitful exploratory avenue for the data. Thus, distributional analyses on reaction time and percent error were added into the analysis of each task to assess the changes in facilitation and inhibition. As a result, these manipulations and analyses should offer further insights into how facilitation and inhibition combine to create congruency effects over different time points in basic conflict task paradigms. For completeness, we also computed standard delta plots by computing the difference between congruent and incongruent trials across the RT distribution, allowing us to observe how changes in facilitation and inhibition effect the overall congreuncy effect.

**Fig. 4:**
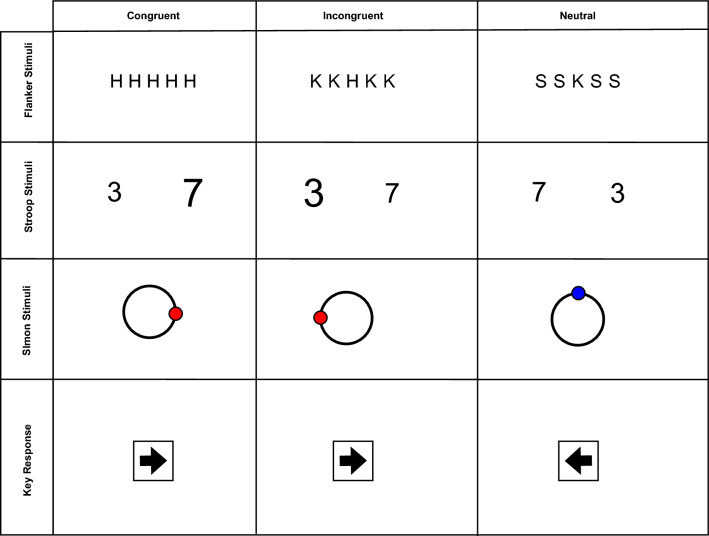
The stimuli for each task are presented across the three experiments, with the linguistic flanker task in the top row, the numeric Stroop task in the second row, and the symbolic Simon task in the third row. The corresponding response key is placed in the bottom row, with the three conditions changing between the columns

## Experiment 1 - Speed-Accuracy Trade-off Flanker Task

Experiment 1 used a letter flanker task with a SAT paradigm. As such, the participants responded to a target letter (K or H) that was flanked by two distractor characters on each side. Three different congruency settings were used, congruent when the flanking stimuli signaled the correct response, neutral when they signaled neither response, and incongruent when the opposing response was signaled. On top of this task, speed-accuracy focus shifted between three settings, resulting in different response patterns by participants. The participants either responded quickly with a short valid response cutoff, accurately with a delayed cutoff, or a mix of both with a cutoff balanced between the two. Blocks of trials were randomized, as were the presentation of trials to counter possible ordering effects. Delta functions for inhibition and facilitation were computed for each speed condition.

### Methods

#### Participants

Participants were recruited from the University of Tübingen campus via an advertisement on the internal mailing system and the SONA system. The experiment was run in person on a computer within the lab. We utilized a sequential sampling paradigm to recruit our participants, namely the independent segments procedure (Miller & Ulrich, 2021). In it, we set up weak and strong boundaries ($\alpha _{weak} = 0.207$, $\alpha _{strong} = 0.001$) with our pilot effect size of $\eta ^2 = 0.271$. We collected participants in batches of 25, with 75 being the absolute cutoff. After running the initial batch of 25, we passed our strong value, thus ending the sampling required. However, as one of the participants did not respond at all, another was collected. Of the 26 that were recruited, one was excluded due to a lack of correct responses (percent correct < 75%). The final total, then, was 25 participants (14 female, 11 male, 0 other, mean age = 23.68 years, SD = 3.20 years). Each received a base rate of approximately €10 per hour, with the experiment lasting around 30 min.

#### Stimuli

The experiment was designed in PsychoPy (Peirce et al., 2019), with the stimuli characters created in Gimp (The GIMP Development Team, 2019). For each stimulus set, a string of five Sans-Serif characters was shown in the center of the screen (Fig. 4). Each string consisted of a central character as the target (H or K) and two flanking distractor characters to the left and right. Three different conditions (congruent, incongruent, and neutral) altered the character strings. For the congruent condition, the flanking characters and target were the same (e.g., HH H HH). The incongruent condition had flanking characters signaling the opposing response (e.g., HH K HH). Finally, the neutral condition had flanking images devoid of a meaningful signal (e.g., SS K SS). All sizes of the stimuli were done in units of ′height′ scaling within PyschoPy. This was done as the latter experiments also utilized said scaling, and consistency was desired. Each stimulus was presented in a square that was 1/50th of the screen size; character size set at (0.02, 0.02). The horizontal string had a distance of 0.02 of the screen size between each image. The position, then, of each character was at (0.0401, 0), (0.0201, 0), (0, 0), $(-0.0201, 0),$ and $(-0.0401, 0)$. Thus, the entire stimulus string was scaled to around 0.1002 of the screen size in horizontal size, and 0.02 in vertical height. Finally, the size of the fixation cross which proceeded the trials was sized at (0.01, 0.01).

#### Procedure

Upon starting the experiment, the participants were presented with the instructions for the flanker task. The stimuli were described as being present in the center of the screen in strings of five characters, with the goal being a single response to the central target. The differing target characters, in line with the original flanker task, were horizontally flanked by two distractors on each side. Three conditions are presented: the congruent condition is a string of the same letter (e.g., KK K KK), the incongruent condition is a string with the central character being flanked by characters for the other response (e.g., HH K HH), and the neutral condition has the central stimulus flanked by a letter with no response mapped to it (e.g., SS K SS). Examples were shown in the instructions. The responses were to press the left arrow button with the left index finger if the target stimulus was K, and the right arrow button with the right index finger if the stimulus was H. Afterwards, the participants completed 60 practice trials.

The practice trials and test trials were identical in terms of the sequence. First, a fixation cross appeared on the screen for 500 ms. Then, after another 500 ms, the stimulus was presented. Participants responded to the stimulus, and their response time and accuracy were recorded. If the participant did not respond correctly, they would receive feedback that their answer was wrong (“Falsch” in red for 350 ms). If the participant did not respond within the 2500 ms allotted to them, they would also be notified that they were too slow (“Zu langsam” in blue for 350 ms).

After the practice round, the participants’ average RT was calculated. Three different time limits were created for the three conditions (Mean RT - SD for Speed, Mean RT + SD for Both, and Mean RT + 3$\cdot$SD for Accuracy). Participants were then presented a screen that explained that three different response patterns would be requested: to “Respond as quickly as possible”, to “Respond as accurately as possible”, or to “Respond as quickly and accurately as possible” (“Antworten Sie so GENAU wie möglich”,“Antworten Sie so SCHNELL wie möglich”, and “Antworten Sie so GENAU und SCHNELL wie möglich”) These relate to the Speed-Accuracy Trade-off, with each instruction set enforcing the prior made time cutoff. After reading the instructions, the main experiment consisted of 54 trials per speed condition, with 10 repetitions overall (Total of $54 \times 3$ trials, or 1620 trials). At the start of each speed response-block, the participant was given instructions on how to respond, depending on the condition. The speed condition was picked at random, with the participants receiving different response cutoffs depending on the response focus and their reaction times. If the participant exceeded the cutoff for a stimulus, the response would be recorded but they were notified that they were too slow. These time cutoffs were adjusted depending on the accuracy rate for the trial set, with a desired accuracy of 97.5% for the Accuracy condition, 82% for the Balance condition, and 65% for the Speed condition. Upon finishing the last of the trials, the participants were instructed to go to the experimenter and report their conclusion of the experiment.

**Fig. 5:**
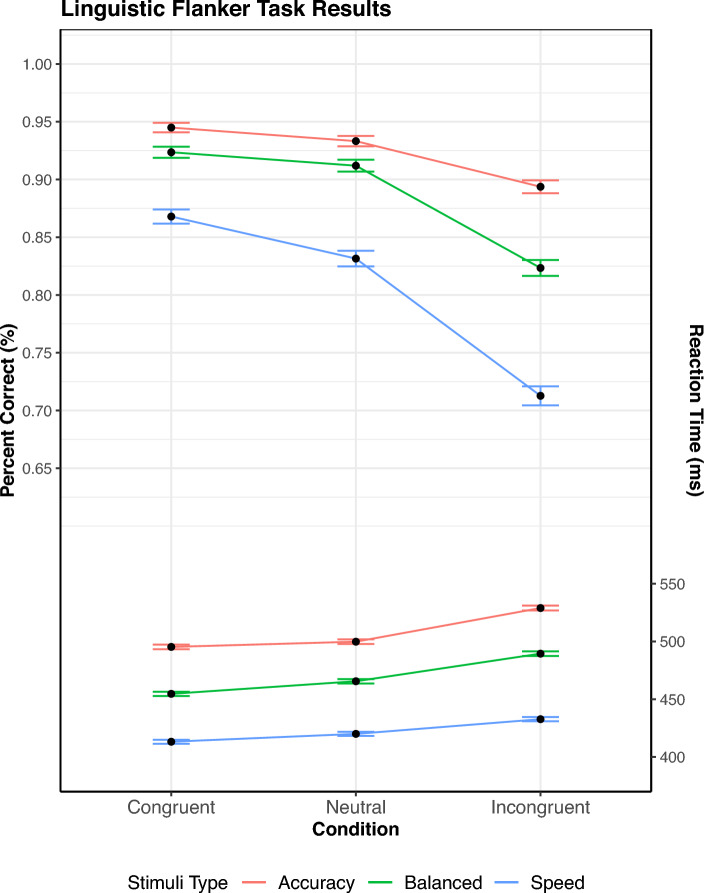
SAT Flanker task: percent correct and mean RT as a function of congruency and stimulus type. Mean RT is shown on the bottom, percent correct is on upper half. The error bar shows $M \pm SE$

**Fig. 6:**
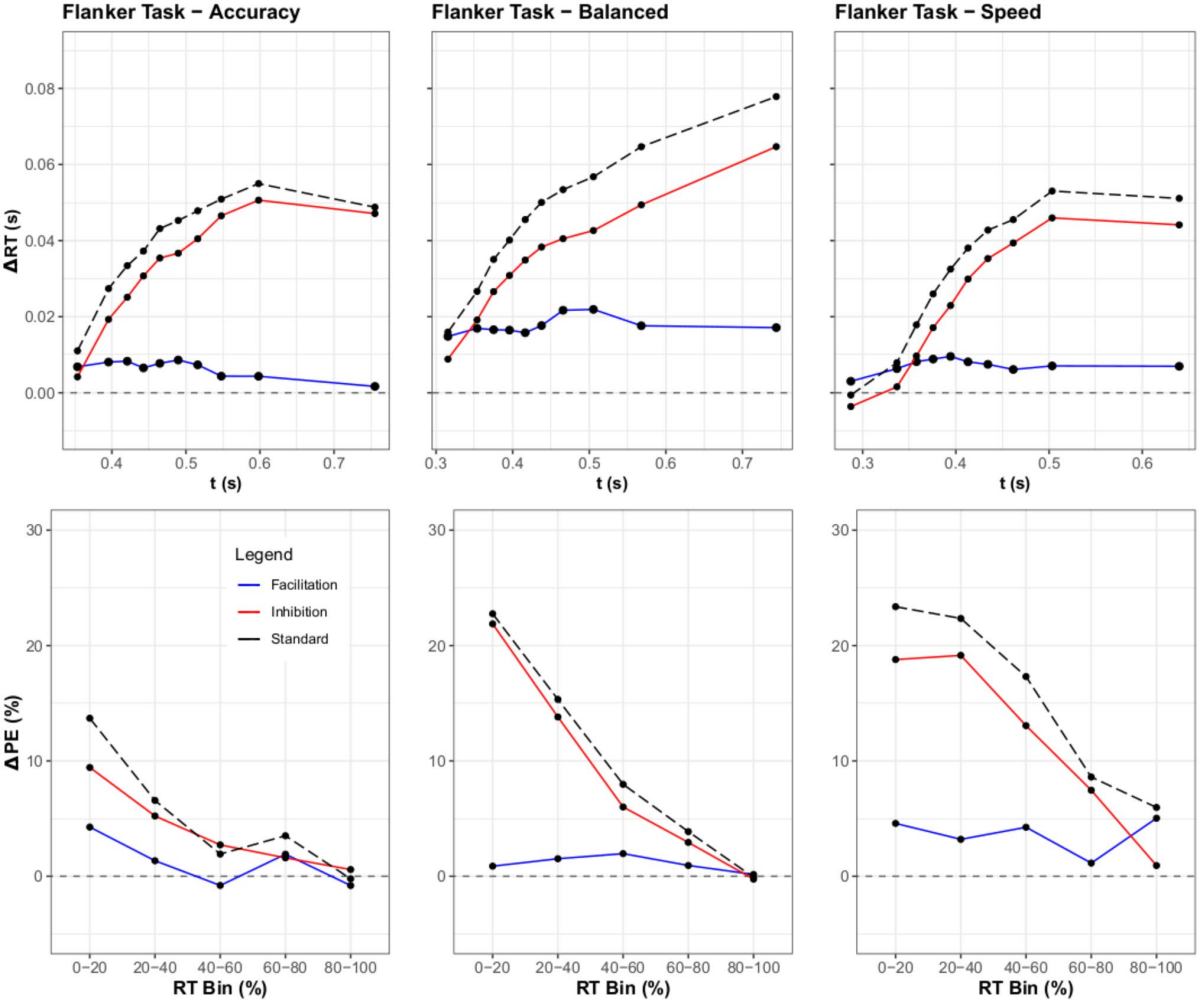
Top Row: Inhibition and facilitation delta plots for the flanker task. It is notable that an increasing dominance of inhibition was seen across the response focus conditions, while facilitation displayed either a stable, low effect, or a decreasing effect within a given response focus condition. Bottom Row: Percent error delta functions for the flanker task were added for completeness, and show an increasing effect of congruency on response focus. This is expected as faster responses often show lower accuracy.

### Results

#### Data analysis

Trials were kept regardless of RT due to the differing conditions in response expectations. Responses in succession that were less than 150 ms were flagged as the participant not following instructions, and were evaluated. Participants who continued this pattern were removed from the study. Only trials with correct responses were analyzed (accuracy: $92.4\%$, balance: $88.6\%$, speed: $80.4\%$). A repeated-measures ANOVA with congruency (congruent versus neutral versus incongruent) $\times$ response focus (accuracy focus versus balance focus versus speed focus) was performed on percentage correct (Fig. 5). The results of this analysis were significant for both response focus [$F(1,48) = 111.26$, $p <.001$, $\eta ^2 =.99$] and congruency conditions [$F(2, 48) = 96.36$, $p <.001$, $\eta ^2 =.80$]. The interaction between them also proved significant [$F(4, 96)=23.01$, $p <.001$, $\eta ^2 =.49$], reflecting larger differences between the congruent, neutral, and incongruent conditions with an increased focus on response speed.

Regarding mean RT, a repeated-measures ANOVA on congruency $$\times$$ response focus with regards to reaction time was performed. The individual effects proved significant as well, with effects on both congruency [F$$(2, 48) = 97.68$$, $$p <.001$$, $$\eta ^2 =.80$$] and response focus [F$$(2, 48) = 115.71$$, $$p <.001$$, $$\eta ^2 =.83$$]. Notably, there was a significant interaction between the two as well [F($$4, 96) = 8.35$$, $$p <.001$$, $$\eta ^2 =.26$$]. In particular, either the inhibitory flanker effect (incongruent − neutral) increased, or facilitation effect (neutral − congruent) decreased.

#### Distributional RT analysis

To assess the resulting differences between the RT distributions of the SAT experiments, RT percentiles were computed for each participant via ten bins ($5\%$, $15\%$, $25\%$,...,$95\%$) across the response focuses and congruency conditions. Then, a three-way ANOVA over Response Speed Focus, Congruency, and Percentile was performed. For this ANOVA, the main effects of response focus, [*F*(2, 48) = 116.41, $p <.001$, $\eta ^2 =.44$], Congruency, [*F*(2, 48) = 100.90, $p <.001$, $\eta ^2 =.14$], and Percentile, [*F*(9, 216) = 197.99, $p <.001$, $\eta ^2 =.84$] were all significant. Notably, the three-way interaction between responce focus $\times$ congruency $\times$ percentile was also significant, [*F*(36, 864) = 1.44, $p =.046$, $\eta ^2 =.002$], adding further merit to the differences found in the ANOVA over base RTs. Thus, the response focus did significantly affect the delta functions, implying a difference between the inhibition and facilitation as well. This can be seen in inhibition and facilitation graphs for the conditions (Fig. 6). The facilitation curves were generally either stable or decreasing, whilst the inhibition curves generally increased over time. Furthermore, inhibition changed the most due to the SAT manipulation, as this increase was genereally less for the balanced condition when compared to the speed and accuracy conditions. This was not expected from either of the models depicted in Fig. 3.

#### Distributional error rates

The distributional error rate for each condition was also computed for the sake of completeness and exploration. Similarly to the distributional analysis of RT, subjects’ responses were grouped by RT in bins across all conditions. Utilizing 5 bins (centered at $10\%$, $30\%$, $50\%$,..., and $90\%$), both correct and error trials were collected, with the mean error rate computed per bin. Then, a three-way ANOVA over Response Speed Focus, Congruency, and Percentile was performed. For this ANOVA, the main effects of response focus, [*F*(2, 48) = 111.52, $p <.001$, $\eta ^2 =.33$], congruency, [*F*(2, 48) = 96.38, $p <.001$, $\eta ^2 =.27$], and percentile, [*F*(4, 96) = 43.00, $p <.001$, $\eta ^2 =.28$] were all significant. Notably, the three-way interaction between response focus $\times$ congruency $\times$ percentile was also significant, [*F*(16, 864) = 1.88, $p <.05$, $\eta ^2 = 0.02$], which coincides with the results of the prior ANOVA on mean accuracy. Upon plotting the delta plots of the distributional error rates for inhibition and facilitation, a consistent pattern of larger differences in percent error for inhibition comparatively to facilitation are clearly seen. Unsurprisingly, percent error appears to decrease with accuracy stress.

#### Discussion

The results of the first experiment provide some unique insights into the the changes of inhibition and facilitation as the response threshold varies. First, the results show evidence of a dominant effect of inhibition, with both the mean ANOVA and distributional analysis finding significant differences relative to facilitation. This can be seen in mean RT with a shrinking difference in facilitation as speed stress increases, and in the distributional analysis regarding the growing effect of inhibition and decrease of facilitation. Second, the delta functions for inhibition and facilitation show an increasing disparity between the two, reaffirming that inhibition appears to be the driving force behind the flanker task. Third the pattern of facilitation deviated from both model predictions, remaining at low levels rather than increasing to be equal with inhibition. Thus, while the pattern of inhibition seems largely in line with model predictions, the models appear to overemphasize the contribution of facilitation.

## Experiment 2 - Speed-Accuracy Trade-off Stroop Task

Much like Experiment 1, Experiment 2 utilized an identical framework of trials and experimental blocks. For the task, a Numeric Stroop Task was used, with participants responding to the highest value of two numbers on the screen via the left and right arrow keys. For the congruency conditions, the size of the numbers would vary. The condition was congruent if the larger number had the highest value, neutral if both the numbers were the same size, and incongruent if the smaller-valued number was presented in a larger size. As the same SAT setup was used, delta plots for facilitation and inhibition were created once again.

### Methods

#### Participants

Participants were recruited on Prolific. All participants needed to have German as a first language and possess either a QWERTY or QWERTZ keyboard. Similarly, with the Stroop experiment, Prolific’s screening procedure allowed for an appropriate rejection and collection of replacement participants. The sample size was selected using the same procedure as Experiment 1. Thus, the final total was 25 participants (14 females, 11 males, 0 other, mean age = 32.5 years, SD = 10.41 years). Each received a base rate of € 11.93 per hour, with the experiment lasting around 30 min in total.

#### Stimuli

The stimuli consisted of a fixation cross as well as two numbers in Arial font Fig. 4. A number appears on both sides of the fixation cross, sized at (0.15, 0.15) of the screen, at positions $(-0.1, 0), (0.1, 0)$ in units of screen size. The numbers vary in both physical size (e.g., left is larger and right is smaller, right is larger and left is smaller) and numeric distance (e.g., 7 versus 4, 3 versus 6). The variation in size was either 0.075 or 0.1 in units of screen size, and the variation in numeric distance range is from 1 to 4. The size of the stimuli shifted depending on the condition of the trial.

#### Procedure

In order to recruit participants, the experiment was advertised on Prolific as a Speed-Accuracy Tradeoff Stroop experiment, with a description of the task and requirements presented alongside it. The participant then was sent to Sosci Survey in order to fill out proper consent forms. After agreeing to the experiment, the participant was given a link to the experiment on Pavlovia. Upon starting the experiment, the participants were presented with the instructions for the numeric Stroop task. The stimuli were described as being present in the center of the screen as a pair of numbers. The pairs differed in size and numeric distance, with participants needing to respond to their numeric values. Three different conditions (congruent, incongruent, and neutral) altered the numeric pairs accordingly. For the congruent condition, the higher valued number has the largest size (large 7 v small 3). In the incongruent condition, the lower valued number has the largest size (small 7 v large 3). And for the neutral condition, both numbers have the same physical size, but one is larger in value (small 7 v small 6). The responses were to press the left arrow button with the left index finger if the numerically larger stimulus was on the left, or the right arrow button with their right index finger if the numerically larger stimulus was on the right.

Afterwards, the participants completed 72 practice trials. For the individual trials, the structure was kept nearly the same as in Experiment 1. However, out of concern for time and attention, the fixation cross-exposure was reduced to 100 ms, and the stimulus onset was set at 500 ms, rather than the previous 500 and 1000 ms structure. Aside from this shift in time, the overall trial remains consistent.

The procedure after these practice trials is identical to the SAT flanker task, aside from the number of trials and blocks. The participants participated in each speed condition 8 times, with each block containing 72 trials. These were shrunk from the earlier study due to a concern over participant attention.

**Fig. 7:**
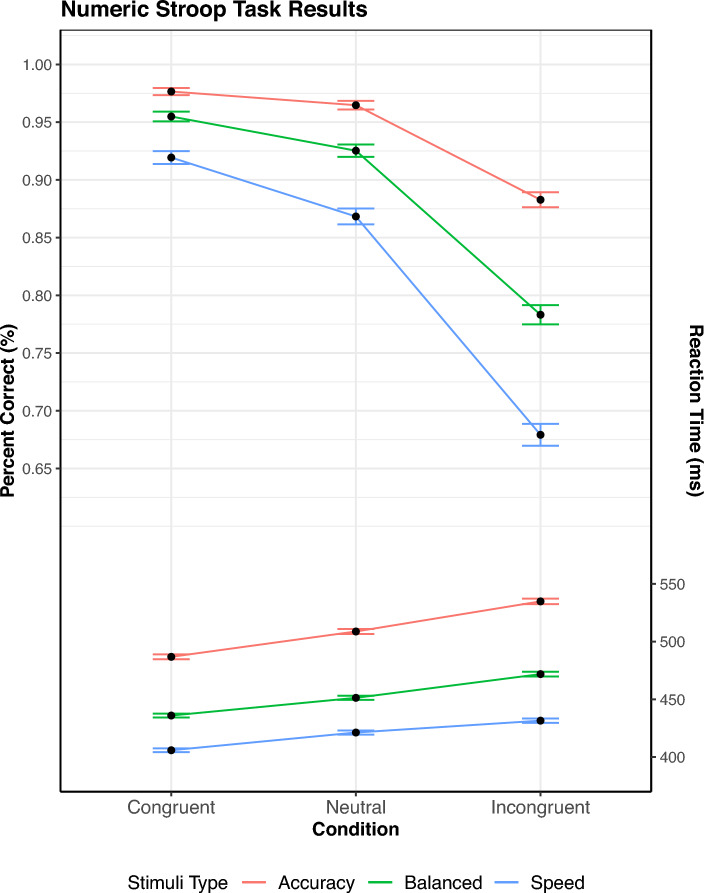
SAT Stroop task: percent correct and mean RT as a function of congruency and stimulus type. Mean RT is shown on the bottom, percent correct is on upper half. The error bar shows $M \pm SE$

**Fig. 8:**
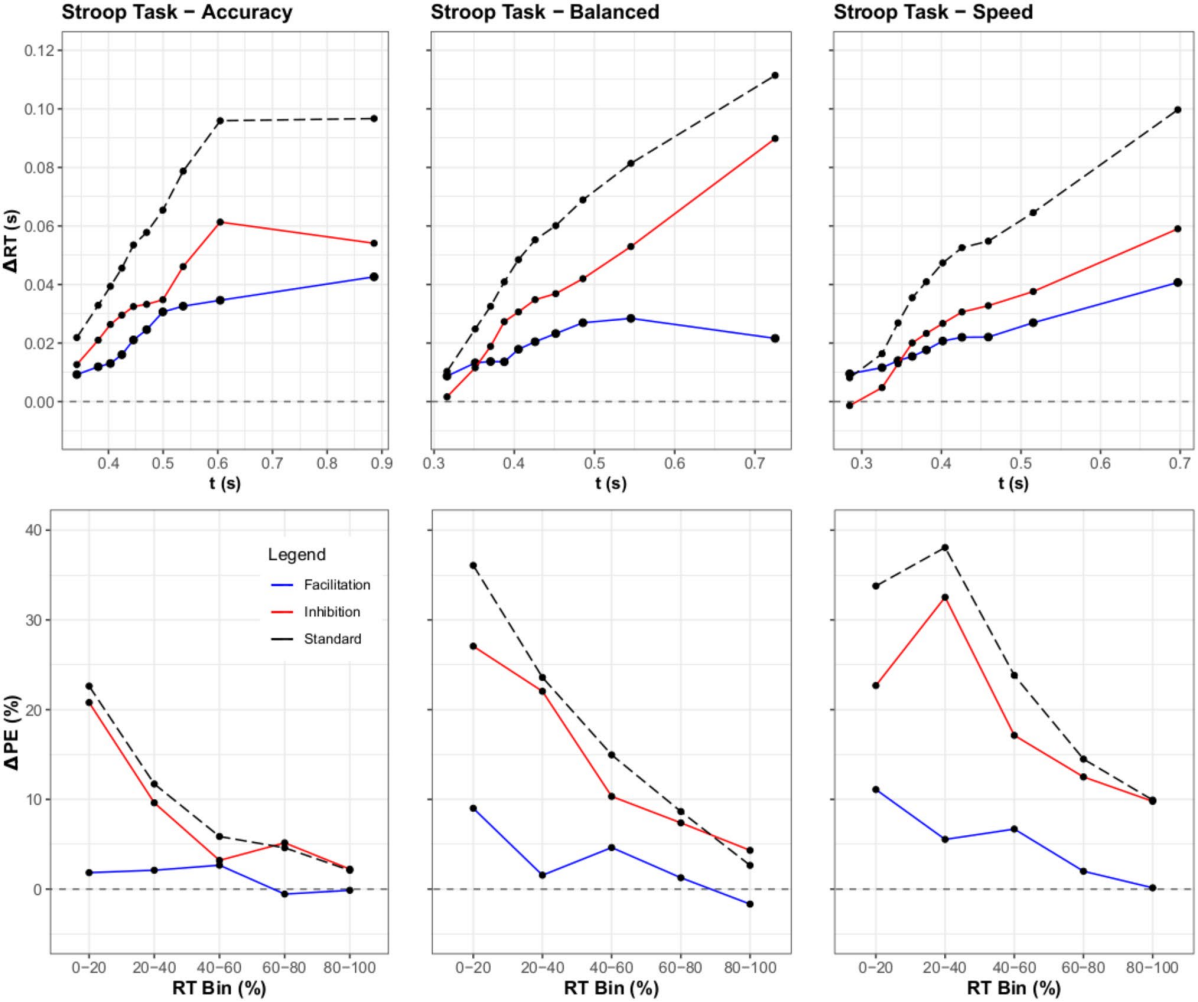
Top Row: Inhibition and facilitation delta plots for the Stroop Task are displayed. While the tail behavior appears to shift significantly for facilitation, the overall pattern of inhibition and facilitation remains similar across the response speed conditions. Bottom Row: The delta function for percent error is added for completeness once again, revealing similar effects as in Experiment 1. The means for binned RT are along the x-axis, with change in percent error along the y-axis.

### Results

#### Data analysis

**Fig. 9:**
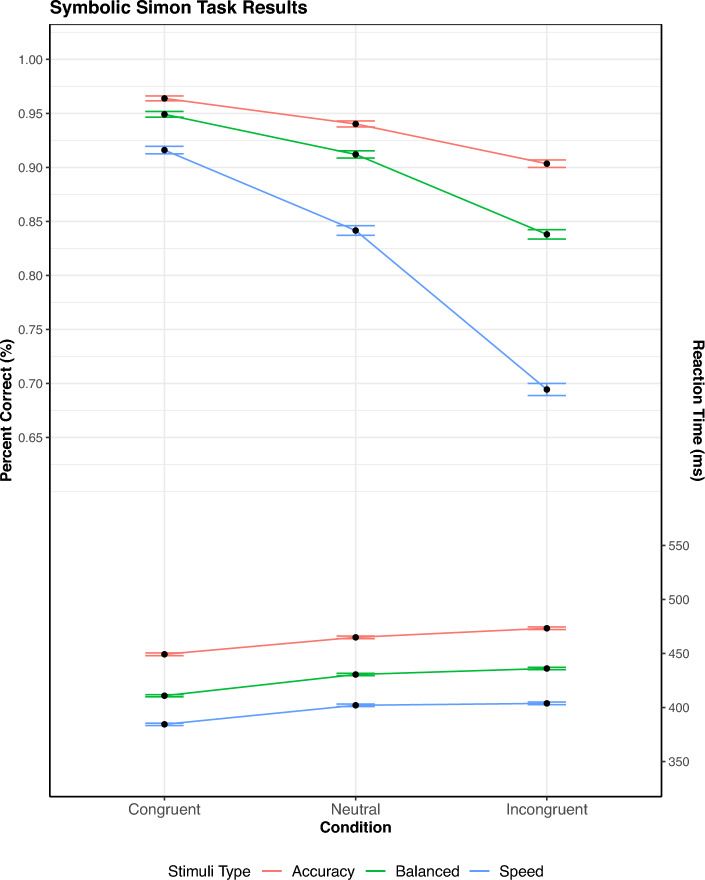
SAT Simon task: percent correct and mean RT as a function of congruency and stimulus type. Mean RT is shown on the bottom, percent correct is on upper half. The error bar shows $M \pm SE$

Following the example of Experiment 1, trials were kept regardless of RT, and responses were flagged in the same manner as before. Correct responses were analyzed (accuracy: $94.1\%$, balance: $88.8\%$, speed: $82.2\%$) A repeated-measures ANOVA was once again performed on congruency $\times$ response focus regarding percentage correct (Fig. 9). The results of this analysis were significant effects for both response focus [$F(2, 48) = 92.09$, $p <.001$, $\eta ^2 =.79$] and congruency conditions [$F(2, 48) = 263.09$, $p <.001$, $\eta ^2 =.92$ ]. The interaction between them also proved significant [$F(4, 96) = 25.74$, $p <.001$, $\eta ^2 =.52$], which makes sense considering the increasing error rate associated with speed on the incongruent condition.

Similarly, a repeated-measures ANOVA for RT regarding congruency $\times$ response focus was also run. The individual effects proved significant as well, with effects on both congruency [(F(2, 48) = 136.56, $p <.001$, $\eta ^2 =.85$] and response focus [$F(2, 48) = 45.76$, $p <.001$, $\eta ^2 =.66$]. As in Experiment 1, there was a significant interaction between the two as well [$F(4, 96) = 6.84$, $p =.001$, $\eta ^2 =.22$].

#### Distributional RT analysis

To assess the resulting differences between the RT distributions of the SAT experiments, RT Percentiles were calculated per participant similarly to the first experiment. Significant findings were again obtained for the main effects of response focus, [*F*(2, 48) = 45.78, $p <.01$, $\eta ^2 =.38$], congruency, [*F*(2, 48) = 136.63, $p <.001$, $\eta ^2 =.16$], and percentile, [*F*(7, 168) = 1739.14, $p <.001$, $\eta ^2 =.73$]. Contrary to the pattern of Experiment 1, the three-way interaction between response focus $\times$ congruency $\times$ percentile was non-significant, [*F*(28, 672) = 0.68, $p = 0.90$, $\eta ^2>.001$]. From these results, one can intuit that while each component contributed to some change in inhibition and facilitation, these changes did not significantly interact. For the Stroop task in contrast to the flanker task, Fig. 8 shows that facilitation and inhibition tend to increase with time. This follows Experiment 1’s results as facilitation shows a larger slope than before, but not as large as inhibition.

#### Distributional error rates

Similarly to Experiment 1, the distributional error rates for each condition were computed. Subject responses were binned in the same manner as in Experiment 1, with 5 bins for ordinal RT across all conditions. Another three-way ANOVA over Response Speed Focus, Congruency, and Percentile was performed. For this ANOVA, the main effects of response focus, [*F*(2, 48) = 91.72, $p <.001$, $\eta ^2 =.31$], Congruency, [*F*(2, 48) = 264.02, $p <.001$, $\eta ^2 =.50$], and percentile, [*F*(4, 96) = 101.85, $p <.001$, $\eta ^2 =.41$] were once again all significant. Similarly to Experiment 1, the three-way interaction between response focus $\times$ congruency $\times$ percentile was also significant, [*F*(16, 384) = 4.29, $p <.001$, $\eta ^2 = 0.04$]. The plotted inhibition and facilitation accuracy curves show a similar result to Experiment 1 as well, with a notable decrease in error as the focus on accuracy increases.

### Discussion

The results of Experiment 2 are similar to the first experiment, however, some notable differences arose. Much like Experiment 1, the interaction of response focus is clearly shown by the ANOVA results, with the ANOVA of mean RT and PC showing significant results. However, the shift in response patterns was not as clear as what was observed with the linguistic flanker experiment. Regarding the ANOVA on RT, while a significant difference was observed overall, these results conflicted with the non-significant results found in the distributional analysis. The cause of this problem can be more readily observed in the facilitation and inhibition curves (Fig. 8). Unlike Experiment 1, facilitation appears to increase with accuracy stress, resulting in a gradual balancing of the two effects. The presence of facilitation dominance seen in early RTs and a lower amount of overall facilitation in Speed/Balanced trials may be the cause of the difference between the ANOVA’s. The results appear more in line with SSP and DMC’s predictions, with a gradual increase in facilitation observed.

## Experiment 3 - Speed-Accuracy Trade-off Simon task

As with the other experiments, Experiment 3 has an identical structure in terms of trial blocks and conditions. The experiment utilized a dot Simon task paradigm, with participants responding to the color (red or blue) of a dot with the left and right arrow keys. In an attempt to reduce the effect of foveal processing on the stimuli, a ring was placed in the center of the screen, with the dot appearing around it. For the congruency conditions, the condition was congruent if the response associated with the color of the dot matched the dot’s spatial position, neutral if the spatial position was unrelated to the associated response, and incongruent if the spatial position of the dot mismatched with response. The SAT cutoffs and prompts were the same as the other experiments, but due to the differing response from experiments in Smith and Ulrich (2023), the exact change in facilitation and inhibition for each speed condition increased was unknown. Thus, we predicted that the amount of facilitation would grow more prevalent as the response boundaries increased.

### Methods

#### Participants

Participants had the same requirements as in Experiment 2. Similarly, with the Stroop Experiment, Prolific’s screening procedure allowed for an appropriate rejection and collection of replacement participants. The sample size was selected using the same procedure as Experiment 1 and 2. The final total was 75 participants (24 females, 50 males, 1 other, mean age = 33.17 years, SD = 9.63 years). Each received a base rate of € 11.88 per hour, with the experiment lasting around 30 min in total.

#### Stimuli

The stimuli consisted of a ring and a dot appearing on the screen after exposure to a fixation cross (Fig. 4). The fixation cross appeared in the center of the screen, and was sized at (0.02, 0.02) in units of screen size. The ring was sized at 1/10 the screen area (presented in an image module sized at (0.1, 0.1)) and was presented in the center of the screen. A dot, sized at (0.025, 0.025), varied in color (blue or red) and position $(0, 90 ^{\circ }, 180 ^{\circ }, 270^{\circ })$ around the center ring.

#### Procedure

The recruitment of participants was identical to that of Experiment 2. Upon starting the experiment, the participants were presented with the instruction set for the Simon task. They were asked to respond to the colored dot with either the left arrow key for a blue dot, or the right arrow key for a red dot. They were given examples of the stimuli which represented different possible states. In the same way as the prior experiments, three conditions were utilized. The conditions were as follows: the congruent condition had the response aligned with the position of the dot (a red dot appearing at the $0^{\circ }$ position, a blue dot appearing at the $180^{\circ }$ position). For the incongruent condition, the response was opposite of the position of the dot (a red dot at the $180^{\circ }$ position, a blue dot at the $0^{\circ }$ position). Finally, the neutral condition occurred when the dot was at a vertical position ($90 ^{\circ }$ or $270 ^{\circ }$). The participants completed 60 practice trials. The procedure after these practice trials is identical to Experiment 2, aside from the number of trials and blocks. The participants participated in each condition 7 times, with each block containing 60 trials.

**Fig. 10:**
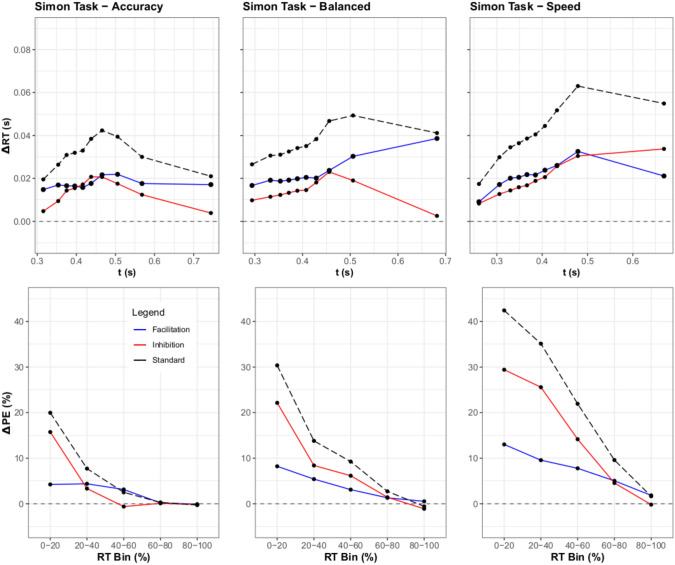
Top Row: Inhibition and facilitation delta plots for the Simon Task are displayed with time in seconds as the x-axis and change in RT is on the y-axis. While the Stroop task showed similar overlap between the two functions, the Simon task exhibits both this overlap as well as significant differences in tail behavior, particularly in the accuracy condition. Bottom Row: The percent error delta functions for facilitation and inhibition is displayed, with change in percent error presented against binned RT.

### Results

#### Data analysis

The exclusion criteria for trials and participants followed the same pattern as the prior experiments. Once again, only correct responses were analyzed (accuracy: $93.5\%$, balance: $90.0\%$, speed: $81.7\%$) A repeated-measures ANOVA on congruency $\times$ response focus was performed on percentage correct (Fig. 9). The results of this analysis were significant effects for both response focus [$F(2, 148) = 187.70$, $p <.001$, $\eta ^2 =.72$] and congruency conditions [*F*(2, 148) = 210.98, $p <.001$, $\eta ^2 =.74$]. The interaction between them also proved significant [$F(4, 296) = 107.00$, $p <.001$, $\eta ^2 =.59$], which makes sense considering the increasing error rate associated with speed on the incongruent condition.

A repeated-measures ANOVA on congruency $\times$ response focus for RT was also run in line with the previous experiments. The individual effects proved significant as well, with effects on both congruency [*F*(2, 148) = 252.28, $p <.001$, $\eta ^2 =.77$] and response focus [*F*(2, 148) = 136.91, $p <.001$, $\eta ^2 =.65$]. Notably, there was a significant yet negligible interaction between the two [$F(4, 296) = 3.45$, $p =.012$, $\eta ^2 =.04$].

#### Distributional RT analysis

RT percentiles were calculated as before. Due to restrictions from the participant data, only eight percentiles could be used ($6.25\%$, $18.75\%$, $31.25\%$,...,$93.75\%$).^1^ Just as before, significant findings were obtained for response focus, [*F*(2, 148) = 135.18, $p <.001$, $\eta ^2 =.30$], congruency, [*F*(2, 148) = 252.71, $p <.001$, $\eta ^2 =.11$], and percentile, [*F*(7, 518) = 616.25, $p <.001$, $\eta ^2 =.81$]. In contrast with the mean RT analysis, the three-way interaction between response focus $\times$ congruency $\times$ percentile was also significant, [*F*(28, 2072) = 2.45, $p <.001$, $\eta ^2 =.002$], in contrast with what was seen for the mean RT values. Thus, the response focus did significantly affect the delta functions, including the patterns exhibited via congruency. Furthermore, the inhibition and facilitation curves (Fig. 10) display differing patterns of dominance regarding facilitation over inhibition depending on response focus. In contrast with the previous experiments, the inhibition delta plots show smaller impacts for slower responses within the balanced and accuracy conditions. For the facilitatory delta plots, there is a a notable increase in slope for slower responses in the balanced condition, with an overall dominant effect across all response speed conditions.

#### Distributional error rates

Following the method of the prior experiments, another three-way ANOVA over response focus, congruency, and percentile was performed on the binned PE and RT data. As with the prior ANOVAs, the main effects of response focus, [*F*(2, 148) = 188.39, $p <.001$, $\eta ^2 =.27$], congruency, [*F*(2, 148) = 211.84, $p <.001$, $\eta ^2 =.28$], and percentile, [*F*(4, 296) = 153.74, $p <.001$, $\eta ^2 =.22$] were significant. In the same vein, the three-way interaction between response focus$\times$ congruency $\times$ percentile was also significant, [*F*(16, 1184) = 10.65, $p <.001$, $\eta ^2 = 0.03$]. The plots of the inhibition and facilitation distributional error rates (Fig. 10) show the same effect of speed focus on percent correct. However, it is interesting that the inhibition error curve drops below the facilitation curve for the accuracy condition, something not as prominently seen in the prior experiments.

### Discussion

Contrary to Experiments 1 and 2, while speed stress did not largely modulate mean RT in Experiment 3, it significantly affected the delta plots. The results display a fairly consistent response pattern, with facilitation dominating across all speed conditions. This result aligns with prior work done in Smith and Ulrich (2023), where the same pattern of mean RTs held across both stimulus sets for the Simon task. In contrast, the distributional analysis had significant results for the inhibition and facilitation curves. When consulting the plots in Fig. 10, it can be seen that facilitation frequently showed a larger effect than inhibition. However, this varied in both the speed and accuracy response focus conditions. This stands in stark contrast with the null results found in the initial analysis of the mean data, with the delta function analysis showcasing a more dynamic change in facilitation and inhibition. As SSP primarily predicts a simple increase in inhibition, these effects were unexpected. However, DMC’s account is also somewhat muddled, primarily due to the frequent dominance of facilitation across the speed conditions of the data, rather the dominant inhibition effect expected for the accuracy response focus condition.

## General discussion

The delta plot analysis of congruency effects has traditionally offered a fundamental window in assessing the time course of conflict processing. By investigating delta plots, one can explore how controlled and automatic factors interact over time as a function of experimental manipulation, providing additional information for conflict processing frameworks that are not apparent when examining mean RT alone. Thus, tools like delta plots allow for interesting insights on the differences in accrual over time between congruent and incongruent conditions, such as the noted negative slope commonly seen in the Simon task (de Jong et al., 1994; Pratte et al., 2010; Schwarz & Miller, 2012). However, by considering only the difference between test trials, it is unclear to what extent the time-course of delta plots reflect facilitatory and/or inhibitory components and how such components can be conceptualized with conflict task models (DMC, SPP). To address this issue, the present study introduced a neutral condition and investigated, for the first time, delta plots separately related to inhibition versus facilitation. To better understand the time-course of these inhibitory- and facilitatory-delta plots, we investigated the SAT effects across three different conflict tasks, as this also allows us to see to what extent the pattern aligns with the predictions of DMC and SSP. Within our discussion, we now elaborate on the insights gained from the delta plot finding as a function of the SAT manipulation against the background of diffusion conflict task models.

As the congruency effect is composed of facilitation and inhibition, the focus of the article was on parsing apart these effects from experimental data, and finding a way to investigate them visually. Drawing upon previous works (Smith & Ulrich, 2023), we implemented a neutral condition in various conflict tasks to observe facilitation and inhibition. Rather than reporting the amount of facilitation and inhibition via a point statistic (such as a mean), we opted to plot the changes of these effects over time via delta functions for each effect. As the general delta plot is linked directly to the congruency effect, and facilitation and inhibition added together compose this effect, a graph showcasing delta functions for facilitation and inhibition allows for a clearer image of how much one effect contributes to the congruency effect over the other. Put simply, if one effect has a higher change in RT or percent error at a time, then it is contributing more to the overall congruency effect. Furthermore, by checking the delta functions at various response focuses, the long-run behavior of these effects could be captured.

Thus to test this approach, we looked into the expectations of two models (Fig. 3) with rather different approaches: SSP and DMC. By simulating from SSP, it was clear that the model predicted high initial amounts of inhibition, with a gradually increasing amount of facilitation. For DMC, the results were more nuanced, with facilitation and inhibition increasing at a similar rate over time. However, both SSP and DMC predicted inhibition as a dominant effect, with facilitation rising to cancel out this dominance over time. While some have argued for the dominance of inhibition (Ridderinkhof, 2002; Ridderinkhof et al., 2004), to our knowledge, no prior studies have demonstrated this using more fine-grained distributional analyses and considering quantitative models.

Thus, the results of our experiments revealed not only the efficacy of plotting delta functions for inhibition and facilitation, but also shed some light regarding the components driving congruency effects. Specifically, in all of the experiments it is clear from the graphs that inhibition drives most of the changes in the standard delta plot. The flanker task showed a negligible contribution of facilitation on the delta plot (Fig. 6), and the Stroop task showed a near constant dominance of inhibition over facilitation. The Simon task was the only task to show prolonged facilitation dominance. However, it is clear from the delta plots that inhibition is a common driver of the congruency effect (the accuracy and balanced conditions in Fig. 10). The reason why the delta plots might differ between the three tasks could be due to the unique conflict loci processed in each task. For example in the Simon task the conflict could be based at the level of response mapping, whereas in the flanker and Stroop task it could be derived from semantic processing.^2^

The facilitation and inhibition curves also provided meaningful insights into mean RT results for the Stroop and Simon tasks. By looking at the mean RT for the Stroop task (Fig. 7), it appears that speed stress has a mild effect on inhibition and facilitation, with the proportion wavering over the different stresses. However, the inhibition and facilitation graph shows that these results come from the simplification of the data, revealing that the basic trends in evidence accrual remain fairly consistent across time stress (Fig. 8). The reverse occurs for the Simon task, however, where the mean RT appears consistent across speed stress (Fig. 9). This starkly contrasts with the inhibition and facilitation curves, where speed stress has a significant interaction with congruency and percentile, which can be clearly seen in how the curves behave (Fig. 10). Thus, the finer-grained information offered by these curves also provides further details for mean RT results.

**Fig. 11:**
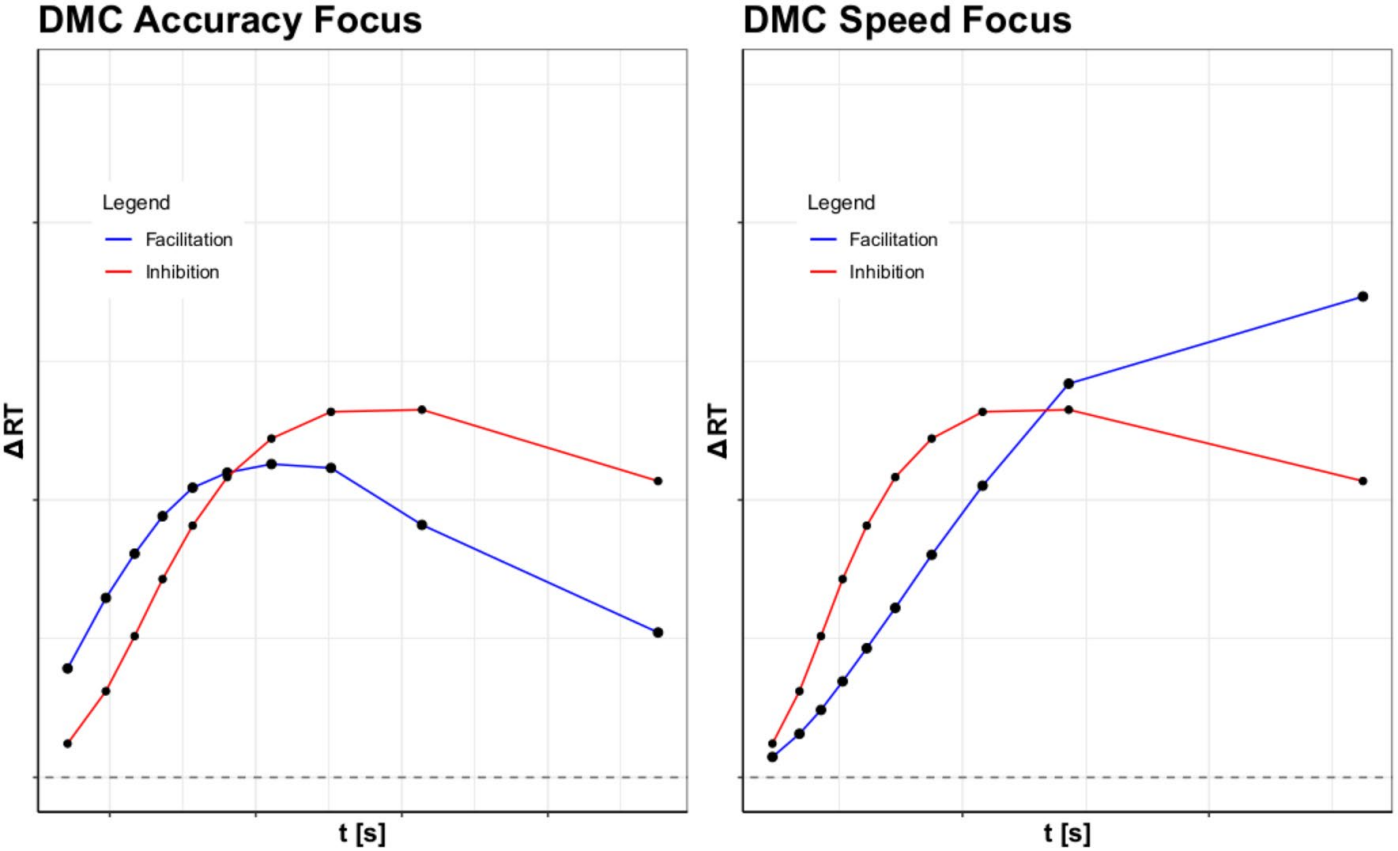
Figure displaying facilitation and inhibition plots for DMC’s prediction for the Simon task with speed condition (left) and accuracy condition results (right). Parameter settings were held constant from the original DMC article (Ulrich et al., 2015). These were generated from 100,000 trial simulations for congruent, neutral, and incongruent conditions. The SAT conditions were obtained via setting the boundary condition for DMC to 34.56 for speed and 74.56 for accuracy. Consult the code resources for further details and scaling.

From this, it is clear that many of the predictions from DMC and SSP hold up to empirical data. Both models predicted the flanker task’s inhibition dominance, with SSP’s prediction of an early inhibition spike being particularly accurate. The Stroop task’s inhibition dominance was also predicted by both SSP and DMC, alongside the shrinking difference between facilitation and inhibition. For the Simon task, DMC also predicts the dominance switch of inhibition and facilitation, with facilitation being dominant most of the time. Thus, a number of the patterns observed within models are seen in the empirical data, bolstering claims about trends in facilitation and inhibition.

However, the empirical data also showed a number of divergences from model predictions, particularly in regards to the expectation of facilitation. Both DMC and SSP assume that as the accrual process continues, the proportion of facilitation to inhibition will become equal. Thus, facilitation is commonly expected to rise, while inhibition either falls or stagnates. However, in the flanker and Simon tasks, we see evidence on the contrary. The flanker task, which SSP was designed for, shows very little increase in facilitation across response focus, and at times even a decrease. This is not restrained to SSP, however, as DMC also predicts a steady convergence of the two delta functions.

In regards to DMC, its predictions were also mixed regarding the Simon task. For the Simon task, the model predicts that early speed-focused responses will have a greater inhibition dominance, and the opposite in the accuracy-focused response condition (Fig. 11). However, the results from our experiment show the opposite, with inhibition overtaking facilitation in the speeded condition for later responses. Likewise, the accuracy condition displayed a large decrease in inhibition after an initial spike, but a steady or growing response from facilitation. As DMC assumes the gradual tapering off of congruency effect as the accrual process continues, it is possible that given a sufficiently large boundary setting we would find that facilitation will continue to decrease. Though given the current data, it appears that inhibition would decay first, rather than facilitation, which implies that the shrinking standard delta function is not due to a mutual decrease in inhibition and facilitation, but rather a staged decrease in inhibition with facilitation possibly following.

As this article was focused on presenting the facilitation and inhibition curves as potential tools, there are numerous avenues for further development. Due to our focus on experimental evidence, a thorough exploration of possible facilitation and inhibition curves from various models could prove useful to check their validity and for possible improvements. Another study (Martinon et al., 2024) also endeavored to further parse apart the types of conflicts that make up these individual inhibition/facilitation effects, making it feasible that these graphs could be decomposed further if given the proper theoretical motivation. On the more experimental side, the use of different stimuli to check for variation in inhibition and facilitation patterns could be interesting as both a robustness check and to see if such a small change could have a high impact on how congruency effects come about. These graphs also could be implemented to check the validity of current methods of increasing or decreasing inhibition, as decreases in facilitation can also account for the same effect in congruency effect measures. Thus, it is the authors’ opinion that these curves could prove diagnostic for both models and methods for congruency.

Finally, the differences between the curves lead to some troubling questions regarding the controlled and automatic process. For most models, the amount of automatic activation is presumed to be equal for both the congruent and incongruent conditions. However, the disparity in facilitation and inhibition witnessed in the flanker task could be seen as evidence on the contrary. Thus, there are two ways of interpreting these results: one via the automatic process and the other with the controlled process. The automatic account of this difference is that of Evans and Servant (2022), where different amounts of automatic activation are used for congruent and incongruent trials. However, their account does not address the question of how the brain can anticipate and pre-allocate this activation before a trial. On the other hand, the controlled process account states that, rather than acting as a simple straight line or midpoint (Smith & Ulrich, 2023), the controlled process may be more dynamic than expected. As such the odd imbalances may be a result of a dynamic evidence accumulation of the controlled process.

In conclusion, the results of our experiments show that inhibition and facilitation can vary widely over the time course of evidence accrual, contributing in varying degrees to the congruency effect. The inhibition curves seen in our experiments also support the hypothesis that inhibition drives the congruency effect as theorized in Ridderinkhof (2002). By gathering higher fidelity information, the causes of effects seen in mean RTs and PE are further explained, allowing for more in-depth analysis of the mechanisms at hand. This information also proves useful in exploring model behavior, with a variety of predicted behavior being supported and violated. Thus these curves could serve as a diagnostic tool for models, as well as for experiment piloting and implementation.

## Data Availability

All data and code can be found at the OSF and Github links provided in the title.

## Shrinking spotlight model

For SSP, the amount of influence a specific area has over the selection process can be written as:

1 $$\begin{gathered} a_{{outer}} (t) = \int_{{ - \infty }}^{{ - 0.5}} \phi (x{\mid }0,\sigma _{a} (t))dx, \hfill \\ a_{{central}} (t) = \int_{{ - 0.5}}^{{0.5}} \phi (x{\mid }0,\sigma _{a} (t))dx, \hfill \\ \end{gathered}$$

where $a_{outer}$ is the influence of the flankers, $a_{central}$ is the influence of the target, and $\sigma _{a}(t)$ is the function that describes the change in influence as time progresses. Additionally, the quantity $\sigma _{a}(t)$ is defined as:

2 $$\begin{aligned} \sigma _{a}(t) = \max (\sigma _{a} - r_{d} \cdot t, 0). \end{aligned}$$

In the equation above, $\sigma _{a}$ is the spread of the attention and $r_{d}$ is the rate at which the spread shrinks, affording less attention to the outer area. As the participant observes the stimulus, it is expected that their attention spread will narrow in towards the target, heavily biasing the drift rate towards the correct response. To obtain the decision path, then, the influence in favor of the flanking stimuli and the target stimuli are added together over each point of time. The function

3 $$\begin{aligned} v(t) = 2\cdot p_{outer}\cdot a_{outer}(t) + p_{central}\cdot a_{central}(t) \end{aligned}$$

expresses this, with the *p* parameter being the sway in favor of either the target or flanker stimuli. For flankers matching the target, $p_{outer}$ is set to 1, for a mismatch, $p_{outer}$ is set to $-1$.

## Diffusion model of conflict

For DMC, the time-dependent drift rate is given by:

4 $$\begin{aligned} v(t) = A\cdot e^{-\frac{t}{\tau }}\cdot \Big [ \frac{t\cdot e}{(a-1)\cdot \tau } \Big ] \cdot \Big [\frac{a-1}{t}-\frac{1}{\tau }\Big ]+\mu _c. \end{aligned}$$

In the equation above, *v*(*t*) stands for the current accrual of information at time *t*, *A* is the amplitude of the automatic function, $\tau$ is a scaling parameter for the automatic function, *a* is the setting for the gamma function’s shape, and $\mu _c$ is the constant drift rate of the controlled process. Similar to the *p* variable in SSP, *A* is set as negative for the incongruent condition, and positive for the congruent condition, representing the automatic activation. The resulting function is flexible, and can account for the observed time-course of congruency effects within Stroop, flanker, and Simon tasks.

## Footnotes

1 The results in the previous two experiments were similar when eight instead of nine percentiles were used.

2 We thank Nabil Hasshim for suggesting this interpretation.
